# *In vitro* activity and *in vivo* efficacy of sulopenem against *Bacillus anthracis* in inhalational anthrax mouse and rabbit models

**DOI:** 10.1128/aac.00399-26

**Published:** 2026-07-17

**Authors:** Jennifer Chua, Annette M. Gray, Steven I. Aronin, Sailaja Puttagunta, Michael W. Dunne, Stephanie A. Halasohoris, Jade L. Spencer, Ashley L. Babyak, Majorie E. Torres, Bobby J. Curry, Christopher P. Klimko, Christopher K. Cote, Mary K. Hourihan, J. Matthew Meinig

**Affiliations:** 1 Bacteriology Division, US Army Medical Research Institute of Infectious Diseases 70051, Fort Detrick, Maryland, USA; 2 Chenega Professional and Technical Service, Frederick, Maryland, USA; 3 Iterum Therapeutics 736555 , Old Saybrook, Connecticut, USA; The Peter Doherty Institute for Infection and Immunity, Victoria, Australia

**Keywords:** ciprofloxacin, biodefense, sulopenem, *Bacillus anthracis*, anthrax

## Abstract

*Bacillus anthracis* is a gram-positive, spore-forming bacterium and the etiological agent of anthrax. As a Tier 1 Select Agent, the pathogen can be isolated from the natural environment, and the endospore is a significant biological threat that can be produced in large quantities, stored, and disseminated by aerosolization. The intentional release of *B. anthracis* spores has the potential for mass casualties and is a serious threat to public and military health. Fluoroquinolones and tetracyclines are common antibiotics used for post-exposure prophylaxis and treatment in the United States (U.S.), whereas penicillin is the drug of choice throughout the rest of the world. However, these commonly used antibiotics may be contraindicated or suboptimal in a public health crisis. Thus, there is a need for novel medical countermeasures to be developed and tested against *B. anthracis*. Sulopenem is a broad-spectrum, orally bioavailable penem antibiotic recently approved in the U.S. for the treatment of uncomplicated urinary tract infections in women with limited therapeutic options. Here, we demonstrate that sulopenem has potent *in vitro* activity against both wild-type virulent *B. anthracis* strains, as well as biosafety level 2 surrogate strains resistant to current standards of care. Using human-equivalent dosing regimens, we show that sulopenem is highly protective in both the mouse and rabbit inhalational anthrax models. Together, these results support continued evaluation of this oral penem as a countermeasure for inhalational anthrax.

## INTRODUCTION

*Bacillus anthracis* is a spore-forming, gram-positive zoonotic bacterium and the etiological agent of anthrax. Naturally found in soil, it is primarily a pathogen of domestic and wild herbivores (1). Endemic across large swaths of Eurasia, Africa, the Americas, and Australia, natural human disease is primarily associated with agricultural occupations. *B. anthracis* infection in humans is generally categorized into four forms of disease: cutaneous, gastrointestinal, injectional, and inhalational. Primarily associated with skin contact with contaminated animal products, cutaneous anthrax is often self-limiting with a mortality rate of ≤2% when properly treated (2). Gastrointestinal anthrax is associated with consumption of undercooked meat from infected animals and remains exceedingly rare in the U.S. Injectional anthrax was more recently described in a limited number of intravenous drug users in Europe (3). Inhalational anthrax is an extremely aggressive form of disease with a case fatality rate between 43% and 90% depending on the course of treatment (4). A complication that can accompany all forms of the disease is anthrax meningitis, which approaches a 100% mortality rate even with treatment and available critical care (5). In all forms, bacterial virulence is due to the combined presence of two plasmids, pXO1 and pXO2, which encode the anthrax toxin components (edema and lethal toxins) and the capsule, respectively.

Anthrax is recognized as a serious biological threat due to the persistence of the endospore under harsh conditions, scalability, and ease of dispersion by aerosolization. In addition, the pathogen can be acquired from the natural environment in endemic areas (6, 7). Within the last five decades, a number of incidents have underscored the nature of the biological threat *B. anthracis* poses. An accidental release from a facility in Sverdlovsk, Soviet Union, in 1979 killed at least 66 individuals downwind and exemplifies the extreme risk aerosolized anthrax spores pose to human health (8). In 1993, a terrorist organization released anthrax spores from a rooftop in Tokyo, but fortunately, the spores used were an attenuated vaccine strain (9). More recently, the Amerithrax incident of 2001 in the U.S. killed 5 of the 22 infected people (10).

Fortunately, antibiotics remain highly effective when given early during disease progression. In the U.S., a combination of commonly prescribed antibiotics, such as fluoroquinolones and tetracyclines (11), in conjunction with antitoxin (11) and vaccination (12), may be given for cases of sporadic outbreak. In developing countries where anthrax is endemic, penicillin is considered a drug of choice because it is effective, readily available, and low in cost. However, up to 10% of *B. anthracis* strains may be penicillin-resistant due to the high-level constitutive expression of the chromosomally derived β-lactamase genes, *bla1* and *bla2* (13–15). In a public health crisis or resource-limited setting, first-line agents may become limited or not effective against antimicrobial-resistant (AMR) strains (11). Additionally, current guidance for post-exposure prophylaxis with inhalational anthrax requires extended dose regimens (≤60 days), and some first-line agents may be contraindicated or not well tolerated by certain populations (11, 16, 17). Therefore, the development and evaluation of novel countermeasures against anthrax are greatly needed.

Sulopenem etzadroxil is an orally bioavailable prodrug of the thiopenem antibiotic sulopenem that was recently approved as a combination with probenecid, an OAT1/OAT3 inhibitor, by the U.S. Food and Drug Administration (FDA) for treatment of uncomplicated urinary tract infection in women with limited or no alternative treatment options (18, 19). The first FDA-approved oral penem, sulopenem, holds significant clinical potential as it can be leveraged in outpatient settings (18, 19). In common with the clinically established carbapenems, it possesses broad-spectrum antibacterial activity against many gram-positive and gram-negative bacterial pathogens (20). Penems are resistant to the activity of plasmid-mediated extended spectrum and chromosomally derived and plasmid-mediated AmpC type β-lactamases, differentiating them from other β-lactams, which are inactivated by these enzymes (21). As a medical countermeasure against anthrax, sulopenem should also be potent against AMR strains of *B. anthracis* that express the functional β-lactamase gene *bla1*, but not *bla2* (13). *Bla1* encodes an Ambler class A β-lactamase with activity against penicillins (22). *Bla2* encodes a class B metallo-β-lactamase enzyme capable of hydrolyzing a large number of β-lactam substrates, including penems (22).

In this work, we demonstrate sulopenem efficacy against *B. anthracis* both *in vitro* and in two animal models of infection. Antimicrobial susceptibility is shown using both virulent wild-type and biosafety level 2 (BSL2) surrogate AMR strains. The efficacy of sulopenem as post-exposure prophylaxis was established in both the inhalational mouse and rabbit models of anthrax. Our results show that sulopenem meets the criteria for further development against inhalational anthrax.

## RESULTS

### *In vitro* activity

The minimum inhibitory concentrations (MICs) of sulopenem against 30 virulent strains of *B. anthracis* were determined using the microbroth dilution method (Table 1). Sulopenem was exceedingly potent *in vitro* (MIC_90_ = 0.015 µg/mL), which is fourfold lower than ciprofloxacin. Using the FDA-recognized susceptibility test interpretative criteria (i.e., breakpoint) for sulopenem with Enterobacterales (≤0.25 µg/mL), 96% of the strains are considered susceptible. The only sulopenem-resistant strain within the panel is SK57 (≥8 µg/mL), which highly expresses Bla1 and Bla2 (23). Excluding SK57, the MIC range for sulopenem was ≤0.004 to 0.03 µg/mL. Using the Clinical Laboratory Standards Institute ciprofloxacin breakpoint for *B. anthracis*, 100% of the tested strains were considered susceptible in the same 30-strain panel (Table 1).

An 11-strain panel of BSL-2 AMR strains in the non-select agent UT308 or Sterne background was curated for this study based on their elevated resistance to penicillin or ciprofloxacin (defined as ≥4-fold increase in MIC values compared to the parental strain) (Table 2). When the panel was tested, 92% of the strains were susceptible to sulopenem compared to 58% that were susceptible to penicillin. Similarly, 92% of strains were susceptible to meropenem. UT308, BA305, BA307, and BA304 were susceptible to sulopenem (breakpoint ≤0.25 µg/mL) but resistant to penicillin (breakpoint ≤0.25 µg/mL). BA301, also called JB034 (24), is resistant to both sulopenem and penicillin. Within the same panel, 27% were susceptible to ciprofloxacin. All strains were susceptible to gentamicin.

**TABLE 1:** Activity of sulopenem and ciprofloxacin against 30 virulent *B. anthracis* strains

Strains	Origin	Sulopenem	Ciprofloxacin
		MIC (µg/mL)	
Vollum1B	USA	0.03	0.06
Ames	USA	0.015	0.06
K1938	Indonesia	0.015	0.06
K5926	India	0.015	0.03
K7038	South Korea	0.015	0.06
SK57	England	>8	0.06
K7978	Namibia	0.015	0.03
Africa 33	South Africa	0.015	0.03
K8091	Norway	0.008	0.06
K4539	France	0.015	0.03
K9724	Unknown	0.015	0.03
SPS00-53	USA	0.015	0.03
BA1086	Unknown	0.03	0.03
Texas 2	USA	0.008	0.06
Colorado	USA	0.008	0.03
V770	Argentina	0.015	0.03
NH	USA	0.015	0.06
Texas	USA	≤0.004	0.03
17T5	Turkey	0.015	0.03
Ohio	USA	≤0.004	0.03
BA0018	Canada	0.015	0.06
BA1024	Ireland	0.015	0.06
BA1007	USA	0.015	0.03
K5135	Pakistan	0.008	0.03
K1887	France	0.008	0.03
K6835	South Africa	0.008	0.03
K2762	France	0.008	0.03
K7665	Turkey	0.008	0.03
K4241	Italy	0.015	0.06
Turkey32	Turkey	0.015	0.03
MIC_50_		0.015	0.03
MIC_90_		0.015	0.06
Sensitivity Breakpoint		≤0.25	≤0.25
Source		FDA Ent.^*a*^	CLSI M45
% Within breakpoint		96	100

^*a*^ Enterobacterales.

**TABLE 2:** MIC summary for sulopenem and other antibiotics against Sterne and AMR *B. anthracis* strains

Strains	Sulopenem	Penicillin G	Amoxicillin/clavulanate	Meropenem	Ciprofloxacin	Gentamicin
	MIC (µg/mL)					
Sterne^*a*^	0.015	0.12	0.06/0.03	0.03	0.06	0.25
UT308^*a*^	0.25	>64	4/2	0.12	0.03	0.12
BA305/BACI385^*b*^	0.008	>64	2/1	0.015	0.03	≤0.03
BA307BACI386^*b*^	0.06	>64	1/0.5	0.12	0.06	0.06
BA301/BACI393^*b*^^,*c*^	4	>64	8/4	1	4	0.12
BA304/BACI396^*b*^	0.25	>64	1/0.5	0.12	8	0.12
BACI389^*d*^	0.008	≤0.03	≤0.03/0.015	0.008	1	0.25
BACI392^*d*^	0.008	≤0.03	≤0.03/0.015	0.015	1	0.5
BACI422^*d*^	≤0.004	0.06	≤0.03/0.015	0.015	8	0.25
BACI423^*d*^	0.015	≤0.03	≤0.03/0.015	0.015	1	0.5
BACI424^*d*^	0.008	≤0.03	≤0.03/0.015	0.015	1	0.25
BACI429^*d*^	0.008	0.06	≤0.03/0.015	0.015	1	0.5
BACI430^*d*^	≤0.004	≤0.03	≤0.03/0.015	0.008	1	1
Sensitivity breakpoint	≤0.25	≤0.25	≤2/1	≤0.25	≤0.25	≤4
Source	FDA Ent.^*f*^	CLSI M45	FDA Strep.^*g*^	FDA Staph.^*h*^	CLSI M45	CLSI M100 Staph.^*h*^
% Within breakpoint^*e*^	92	58	83	92	27	100

^*a*^ Parental strain.

^*b*^ Derivative of UT308.

^*c*^ Also called JB034.

^*d*^ Derivative of Sterne.

^*e*^ Excluding Sterne from percentage calculations.

^*f*^ Enterobacterales.

^*g*^ *Streptococcus pneumoniae.*

^*h*^ *Staphylococcus *spp.

### Murine studies

**Fig 1:**
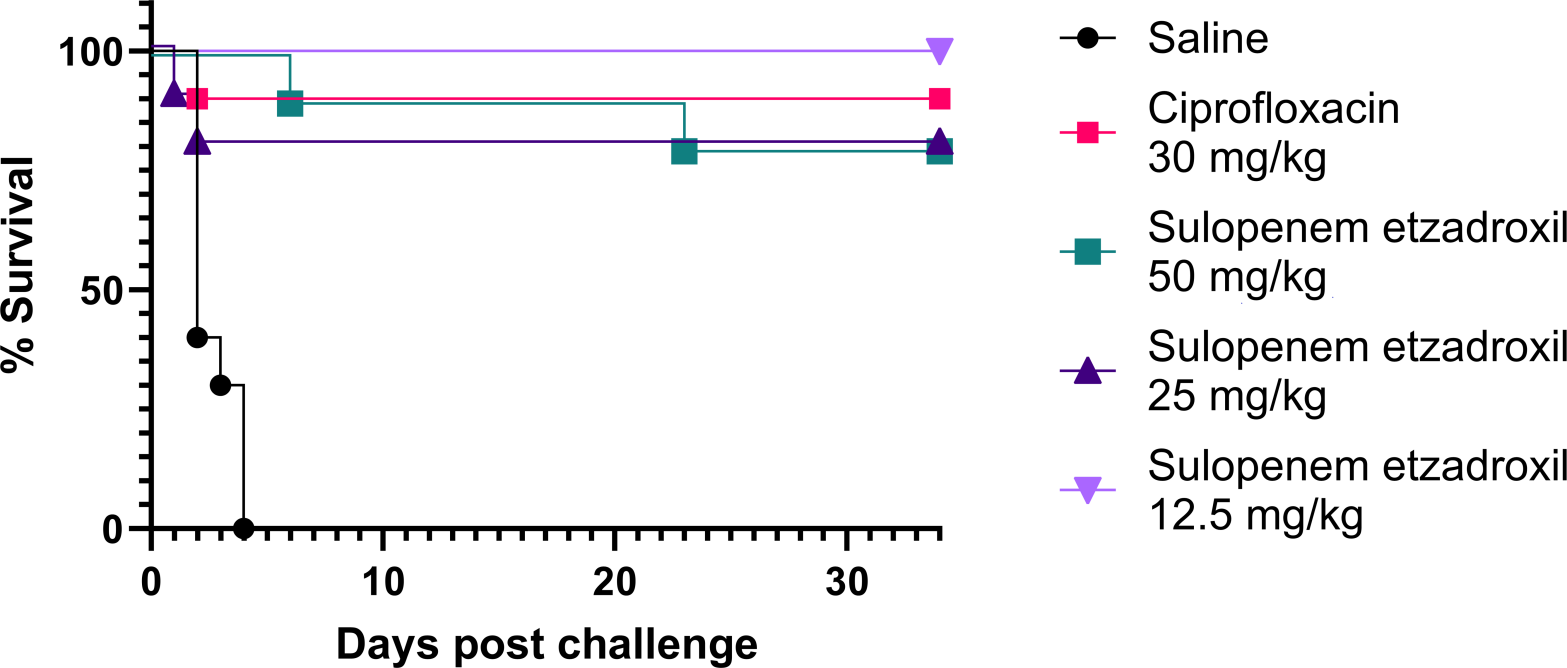
*In vivo* efficacy of sulopenem etzadroxil and ciprofloxacin in a mouse model of inhalational anthrax. Ten BALB/c mice per group were challenged with 15.3 × LD_50_ aerosolized *B. anthracis* Ames spores. Treatment was initiated 24 h post-challenge with sulopenem etzadroxil, ciprofloxacin, or saline and was continued for 14 days. Survival was monitored for 34 days. The percent survival rates for all sulopenem-treated groups were significantly higher (*P* ≤ 0.0013) than that of the saline-treated group.

The efficacy of sulopenem was determined in a murine inhalational anthrax model. Animals were challenged by small particle aerosol with *B. anthracis* Ames spores (mean dose of 15.3 × LD_50_). Treatment was initiated at 24 h post-challenge, given three times a day, and continued for 14 days. Cohorts were monitored to day 34 of the study. Sulopenem was delivered as the oral prodrug sulopenem etzadroxil, whereas the positive control ciprofloxacin was given intraperitoneally. The dose range for sulopenem etzadroxil in the mouse model was predicted to have percent free time above MICs (%T > MIC) based on mouse pharmacokinetic data (data not shown, communicated by S. Aronin, Iterum Therapeutics), in line with those predicted in humans. The challenge dose of *B. anthracis* was uniformly lethal to mice that received saline only, and the median survival time was 2 days, consistent with the established disease model (Fig. 1; Table 3). In contrast, 80%, 80%, and 100% of mice survived until the study termination when treated with 50, 25, and 12.5 mg/kg of oral sulopenem etzadroxil, respectively. All sulopenem-treated groups demonstrated a statistically significant improvement compared to saline alone (*P* ≤ 0.001–0.0013). Survival rates of sulopenem-treated mice were not statistically different from those treated with 30 mg/kg ciprofloxacin (90% survival, *P* = 0.58 vs sulopenem, 50 mg/kg). At the study termination, the lungs and spleens were removed from three mice survivors per treatment group and plated to examine bacterial burden. All spleens in the sulopenem and ciprofloxacin treatment groups were found to be sterile, whereas all the lungs had low levels of bacterial burden (Table 4), indicative of persisting ungerminated spores (25).

**TABLE 3:** Log-rank tests for comparison of sulopenem etzadroxil and ciprofloxacin in mice^*a*^

Treatment	Time of treatment initiation post-infection (h)	No. of dead mice/no. of mice in group	Median time to death (days)	vs saline (*P* value)	vs ciprofloxacin (*P* value)
Saline	24	10/10	2		
Ciprofloxacin (30 mg/kg)	24	1/10	UD	<0.0001	
Sulopenem etzadroxil (50 mg/kg)	24	2/10	UD	<0.0001	0.5842
Sulopenem etzadroxil (25 mg/kg)	24	2/10	UD	0.0013	0.5151
Sulopenem etzadroxil (12.5 mg/kg)	24	0/10	UD	<0.0001	0.3173

^*a*^ UD, undefined.

**TABLE 4:** Bacterial burden in mouse lungs

Treatment	Average CFU/g
Ciprofloxacin 30 mg/kg	1.5 × 10^3^
Sulopenem etzadroxil 50 mg/kg	1.4 × 10^3^
Sulopenem etzadroxil 25 mg/kg	1.9 × 10^3^
Sulopenem etzadroxil 12.5 mg/kg	1.4 × 10^3^

### Rabbit pharmacokinetics

**Fig 2:**
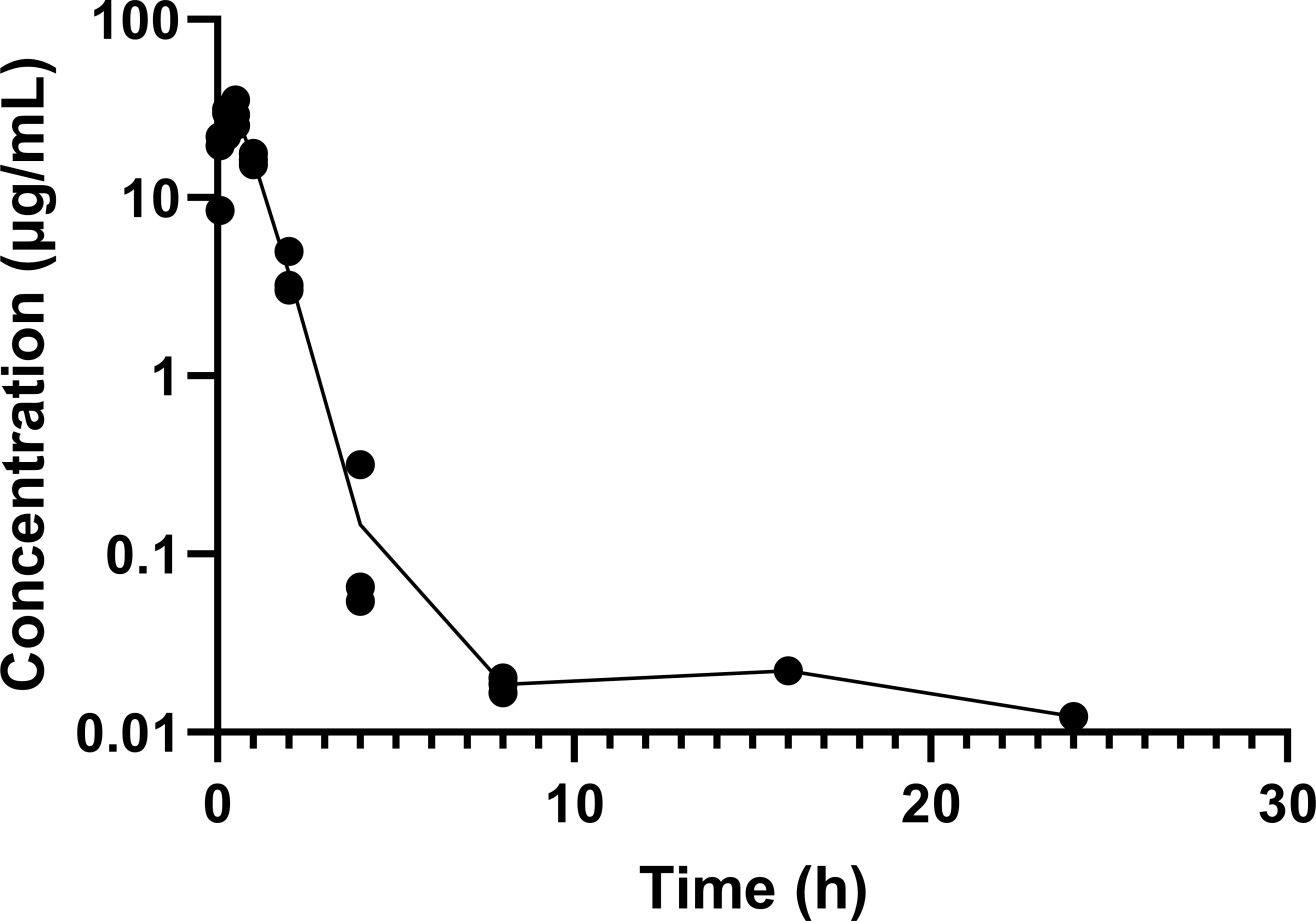
Plasma concentrations over time in naïve NZW rabbits given a single bolus subcutaneous injection of sulopenem (20 mg/kg). Data shown are individual concentrations with a connecting line showing the mean. Data below the limit of quantitation have been excluded from the plot due to the log scale.

To establish a humanized dosing regimen for a rabbit inhalational anthrax study, the pharmacokinetics (PK) of subcutaneous sulopenem in rabbits were determined. The goal was to find a subcutaneous dosing regimen for rabbits that recapitulates important human clinical PK parameters following a 1 g intravenous dose. The subcutaneous route was chosen to produce pharmacokinetics similar to intravenous delivery, but without the need to maintain venous access during extended treatment regimens in a high-containment facility. New Zealand White (NZW) rabbits (*N* = 3) were given a single 20 mg/kg subcutaneous (SC) dose of sulopenem, and serial blood sampling occurred via a venous access port (VAP; Fig. 2). Noncompartmental analysis (Phoenix WINNONLIN, Certara) was used to calculate PK parameters (Table 5). The elimination half-life (*t*_1/2_) averaged 0.89 h, similar to that observed in humans, although the exposure (area under the curve, AUC) was approximately twofold higher than in humans (26). Using experimental data, a one-compartment model was developed to simulate various dose regimens to estimate the pharmacokinetic/pharmacodynamic (PK/PD) index. A subcutaneous 10 mg/kg regimen every 12 hours (q12h) was down-selected, and it was estimated to have a free-time above MIC (%T > MIC) of 70% and 21% for MICs of 0.015 and 1 µg/mL, respectively. The MIC of 0.015 µg/mL corresponds to the sulopenem MIC for *B. anthracis* Ames, which is used for *in vivo* studies, and is in the typical range of penem antibiotics (27). Foulds et al. suggest that the %T > MIC of 15% for a MIC of 1 µg/mL is sufficient for bactericidal activity (26). This target is similar to other findings in a neutropenic thigh infection model, where a target of %T > MIC of ~20% was associated with a 2-log bactericidal effect (28, 29). As these values approximate %T > MIC achievable with both IV and oral dosing of sulopenem, a 10 mg/kg sulopenem (SC, q12h) dose was determined to be a human-equivalent dose in rabbits.

**TABLE 5:** Pharmacokinetic parameters from a single dose of sulopenem in rabbits

	Rabbit (*N* = 3)^a^
Dose (mg/kg)	20
*C*_max_ (µg/mL)	28.2 ± 7.8
*T*_max_ (h)	0.42 ± 0.14
AUC_last_ (µg·h/mL)	37.7 ± 3.0
*t*_1/2_ (h)	0.87 ± 0.08
*V*_*D*_/*F* (mL)	2,331.3 ± 229.6
CI/*F* (mL/h)	1,864.3 ± 142.9

^a^ Data shows the mean and standard deviation from three animals.

### Rabbit efficacy study

**Fig 3:**
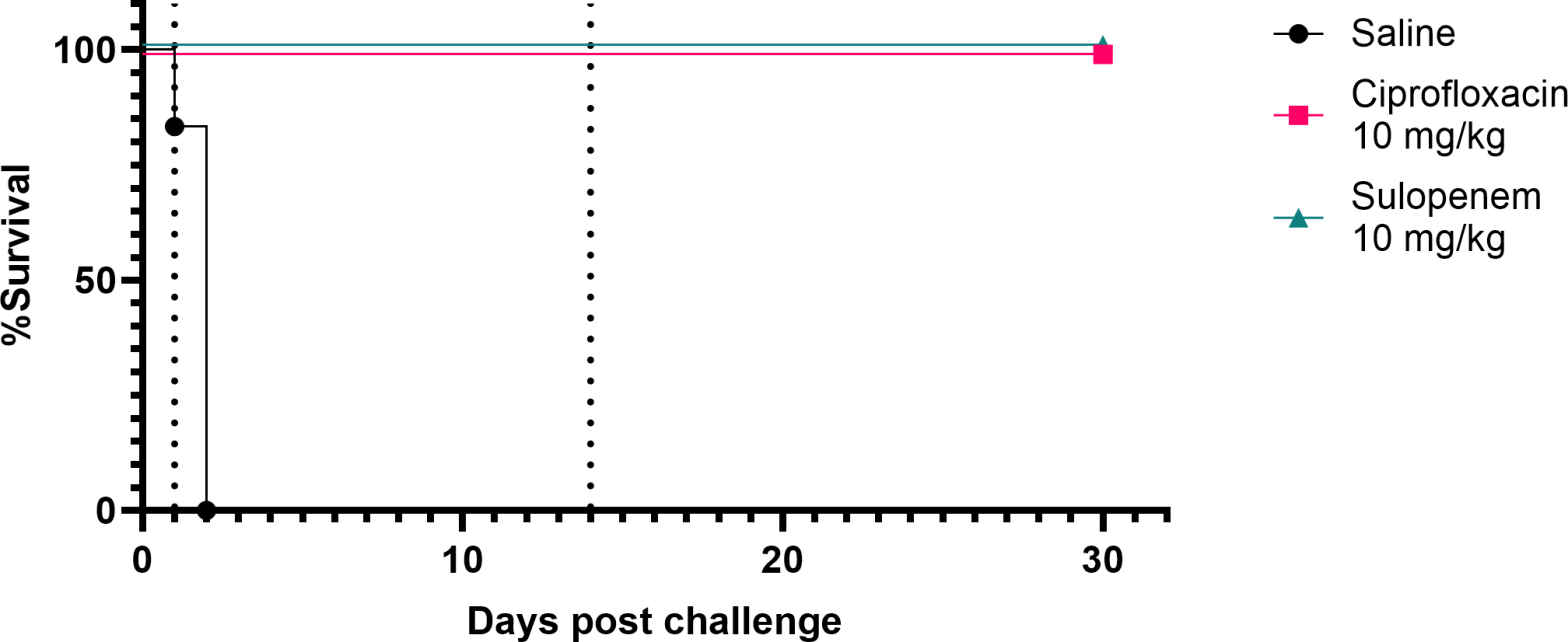
*In vivo* efficacy of sulopenem and ciprofloxacin in a rabbit model of inhalational anthrax. Six NZW rabbits per group were challenged with 41.3 × LD_50_ aerosolized *B. anthracis* Ames spores. The beginning and end of the indicated treatment regimens are indicated by the vertical dotted lines. Survival was monitored for 30 days. Survival was significantly higher in both the sulopenem and ciprofloxacin groups compared to saline (*P* = 0.0012).

The efficacy of sulopenem was assessed in the NZW rabbit model of inhalational anthrax. Three groups of six female rabbits were challenged with *B. anthracis* Ames spores by aerosolization with a mean inhaled dose of 41.3 × LD_50_. Rabbits were treated subcutaneously with saline or human-equivalent doses of sulopenem (10 mg/kg, q12h) or ciprofloxacin (10 mg/kg, q12h) beginning at 24 h post-challenge for 14 days. Animals were monitored for 30 days. All animals in the saline-treated group succumbed to the disease by 48 h post-challenge (Table 6). In contrast, 100% survival was observed in both the sulopenem and ciprofloxacin-treated groups (Fig. 3; Table 6). Survival in both treatment groups was statistically significant compared to the saline control (*P* = 0.0012). In addition, all surviving animals had no detectable bacteremia in the blood at the study termination.

**TABLE 6:** Log-rank tests for comparison of sulopenem and ciprofloxacin in rabbits^*a*^

Treatment	Time of treatment initiation post-infection (h)	No. of survivors/no. in group	Median survival time (days)	vs saline (*P* value)	vs ciprofloxacin (*P* value)
Saline	24	6/6	2		
Ciprofloxacin (10 mg/kg)	24	0/6	UD	0.0012	
Sulopenem (10 mg/kg)	24	0/6	UD	0.0012	1

^*a*^ UD, undefined.

## DISCUSSION

Nearly 40 years have elapsed since sulopenem was first discovered in the laboratories of Pfizer in the 1980s (30). Subsequent development has cumulated into FDA approval of oral sulopenem etzadroxil plus probenecid in 2024 for the treatment of urinary tract infections in women. As the first FDA-approved orally available penem antibiotic, sulopenem has significant potential to combat serious biological threats to both public and military health in an outpatient setting. The goal of this current study was to assess the efficacy of sulopenem in both *in vitro* and *in vivo* models as post-exposure prophylaxis for inhalational anthrax. As a mass casualty scenario involving *B. anthracis* infection may result in serious limitations to inpatient resources, broad-spectrum, oral antibiotics are of particular interest. Sulopenem was highly potent against fully virulent *B. anthracis* (MIC_90_ = 0.015 µg/mL), which is similar to previous reports of other penems (11, 14, 31–33). We have also shown that sulopenem remains active against various fluoroquinolone- and penicillin-resistant strains of non-virulent *B. anthracis*. Penicillin-resistant strains due to high levels of constitutive expression of Bla1 or Bla2 may represent up to 10% of wild-type *B. anthracis* strains; therefore, susceptibility testing of the clinical isolate is recommended prior to initiation of therapy with β-lactams (11, 14, 15). While metallo-β-lactamase Bla2-expressing strains like SK57 may not be susceptible to sulopenem, it may remain an appropriate empiric oral therapy alone or in combination with other antibiotics in mass casualty scenarios. Sulopenem should remain efficacious against Bla1-expressing strains, such as UT308, or strains resistant to standard-of-care antibiotics (Table 2) (34).

For *in vivo* evaluation, the murine and rabbit inhalational anthrax models were chosen as both models have been extensively used in the evaluation of therapeutics against *B. anthracis* (35–45). Sulopenem etzadroxil, the oral prodrug of sulopenem, was highly protective in mice at all doses tested, with 80% survival observed at the highest dose (50 mg/kg). All doses resulted in survival that was not statistically different from treatment with ciprofloxacin. These positive results prompted the evaluation in the rabbit inhalational anthrax model. Using a humanized dose of sulopenem administered subcutaneously, 100% survival was observed in rabbits treated with either sulopenem or ciprofloxacin, while saline-treated rabbits rapidly succumbed to infection (median survival time = 2 days). While high levels of protection were observed in both the mouse and rabbit experiments, one limitation of the current study is the reliance on extrapolating PK/PD indices from unrelated pathogens to infections to *B. anthracis*. Additional studies using a hollow-fiber infection model (46) or *in vivo* dose-ranging studies (47) could provide *B. anthracis*-specific PK/PD targets for future development. Additionally, we have tested both the oral prodrug and active drug in mice and rabbits, respectively. While the oral prodrug sulopenem etzadroxil is currently the only FDA-approved formulation, the choice of testing sulopenem by the subcutaneous route in rabbits was born out of logistical considerations for executing a 14-day treatment regimen in rabbits in a high-containment facility. Further work should examine equivalency between using the intravenous and oral formulations, as well as the potential for an IV-to-oral step-down regimen.

With its proven clinical safety and tolerability profile, combined with *in vitro* potency and efficacy in preclinical models, sulopenem remains an attractive candidate for continued development against biological threats, including anthrax. The ability to leverage the broad-spectrum activity of a penem in an outpatient setting through oral administration would substantially reduce the burden on healthcare staff in a mass casualty scenario while also being more deployable with the warfighter. Given that the generation of drug-resistant mutations to existing oral standards of care has been demonstrated, expanding the medical countermeasure armament to include the first FDA-approved oral penem seems prudent (31, 34, 48–50). Sulopenem remains a promising potential treatment for inhalational anthrax, and these data support its continued development.

## MATERIALS AND METHODS

### Strains and culture

Bacterial strains used in this study are held by the Bacteriology Division or the Biodefense Reference Material Repository at the U.S. Army Medical Research Institute of Infectious Diseases (USAMRIID). A panel of 30 virulent, genetically and geographically diverse strains of *B. anthracis* was utilized for antimicrobial susceptibility testing (33). This panel includes the Ames strain, which was originally isolated from a dead cow in Texas and was used as the challenge strain in subsequent animal models. Of note, SK57 is a penicillin-resistant virulent strain first isolated in England in 1975 (51). The *B. anthracis* BSL2 AMR panel included serially passaged or genetically engineered strains from avirulent, Federal Select Agent Program-excluded backgrounds (UT308 and Sterne). The panel was assembled to include strains with elevated penicillin or ciprofloxacin MICs compared with their parental strains. UT308 is an avirulent derivative of strain 32 and is penicillin-resistant, similar to its parental strain (51). Sterne is a vaccine strain developed in 1935 that encodes, but does not constitutively express, functional *bla1* and *bla2* genes and remains penicillin sensitive (52). All *B. anthracis* strains were grown on sheep blood agar (SBA) plates (Remel, Lenexa, KS) and incubated for 18 to 24 h at 37°C.

Ames spores were generated as previously described (53). Briefly, vegetative bacilli were cultured in Leighton-Doi sporulation medium, purified through Hypague-76 (Nycomed, Princeton, NJ), and washed multiple times in sterile water for injection (WFI) (44, 54, 55). Prior to aerosol challenge, spore suspensions in WFI were heat shocked at 65°C for 30 min (44, 54).

### MIC determination

MICs were determined using microbroth dilution in cation-adjusted Mueller-Hinton broth (CAMHB, Becton Dickinson) according to Clinical Laboratory Standards Institute (CLSI) methodology (56–58). Stocks of sulopenem (Iterum Therapeutics) were prepared in phosphate-buffered solution (PBS, pH 7.4). Stocks of ciprofloxacin, penicillin, amoxicillin, clavulanic acid, meropenem, and gentamicin (United States Pharmacopeia, Rockville, MD) were prepared in accordance with CLSI guidelines (58). Antibiotics were diluted to their corresponding starting concentrations, followed by a twofold dilution series to the final concentration. The final antibiotic concentration ranges following bacterial inoculation were as follows: 8–0.004 µg/mL for sulopenem, meropenem, and ciprofloxacin; 64–0.03 µg/mL for penicillin, amoxicillin, and gentamicin. Clavulanic acid was held at a 1:2 ratio with amoxicillin throughout the concentration series. Quality control strains *Escherichia coli* ATCC 25922, *Pseudomonas aeruginosa* ATCC 27853, and *Staphylococcus aureus* ATCC 29213 were included with all MIC experiments, with results generally within published values. Ninety-six well plates were incubated at 37°C, and MICs were visually assessed at 18 to 24 h. Where available, MIC results were compared to published susceptibility breakpoints (57, 59). As susceptibility breakpoints specific to *B. anthracis* are limited, breakpoints for other gram-positive pathogens with pneumonic indications (*Staphylococcus* spp. or *Streptococcus pneumoniae*) were used as surrogate breakpoints to guide interpretation. As sulopenem has only received an FDA breakpoint for Enterobacterales, this value was used for the interpretation of sulopenem results.

### Drug formulations

The oral prodrug sulopenem etzadroxil (Iterum Therapeutics) was formulated in 0.5% methylcellulose (Sigma). Solutions were prepared at 1.25, 2.5, and 5 mg/mL, corresponding to doses of 12.5, 25, and 50 mg/kg, respectively, in mice. All sulopenem etzadroxil treatments were delivered by oral gavage (0.2 mL/animal). Ciprofloxacin in 5% dextrose (D5W, Sagent Pharmaceutical, Schaumburg, IL) came preformulated at 2 mg/mL and was given as a 0.3 mL injection per animal intraperitoneally (30 mg/kg) (60). Saline (0.9%, Baxter, Deerfield, IL) was delivered by intraperitoneal injection and used as a negative control at 0.2 mL/animal. For the pharmacokinetic study, powdered sulopenem was prepared in PBS and given as a single subcutaneous injection of 20 mg/kg. For the efficacy study in rabbits, sulopenem was reconstituted in saline and given at 10 mg/kg. The humanized dose for ciprofloxacin was determined previously as 10 mg/kg (subcutaneous, q12h) (61). Saline was given as a negative control.

### Pharmacokinetic study

Specific pathogen-free NZW rabbits (~3.5 kg, Envigo) were obtained with implanted jugular VAPs. VAPs were flushed with saline and locked with a taurolidine citrate solution after each blood draw and weekly during the acclimation period after receipt of the animals. Sulopenem was given as a bolus subcutaneous injection. Blood samples were taken from VAPs at 0.083, 0.25, 0.5, 1, 2, 4, 8, 16, and 24 h post-dose and processed for plasma. Plasmas were collected and stored at −80°C until bioanalysis.

Plasma sulopenem concentrations were quantified using tandem liquid chromatography-mass spectrometry (LC-MS/MS). In brief, stock solutions of sulopenem and meropenem were prepared in dimethyl sulfoxide (Sigma-Aldrich, St. Louis, MO) at 5 mg/mL. Meropenem was diluted in 1:1 PBS:methanol (Sigma-Aldrich) to a concentration of 50 µg/mL to make an internal standard working solution (ISWS). ISWS was diluted 1:2,000 in methanol to prepare a crash solution. A nine-point calibration curve and quality control samples of sulopenem were independently prepared by diluting in naïve rabbit plasma. All plasma samples (50 µL) were treated with methanol (100 µL) containing 25 ng/mL of meropenem as an internal standard. Samples were mixed well and clarified by centrifugation prior to analysis of the supernatant by LC-MS/MS. Sulopenem was detected using a Triple Quad 5500 Mass Spectrometry System (AB Sciex, Framingham, MA) in line with a Nexera2/LC30 Prominence UPLC system (Shimadzu, Columbia, MD). Separation was done on a 100 × 2 mm, 4 µm HydroRP column (Phenomenex, Torrance, CA) using a gradient of acetonitrile + 0.1% formic acid in water + 0.1% formic acid over 4 min. Analytes were detected using multiple reaction monitoring with a primary and secondary transition. Sulopenem was quantified as the area-under-the-curve of the analyte peak normalized to the meropenem internal standard, and the standard curve was fit with linear regression with 1/x^2^ weighting. Samples that fall outside the linear range of the calibration curve were diluted in naïve plasma and re-analyzed. The resulting pharmacokinetic data were analyzed by a noncompartmental analysis using WINNONLIN 8.3 software (Certara). A single-compartment model was developed to confirm human-equivalent dose selection in rabbits.

### *In vivo* efficacy models

#### Aerobiology

Small particle aerosols of *B. anthracis* Ames spores were generated using a three-jet collision nebulizer (BGI, Waltham, MA) (62). All procedures were controlled and monitored by the Automated Bioaerosol Exposure System (63). Integrated air samples were obtained from the chamber using an all-glass impinger (AGI) containing sterile water after each exposure. AGI samples were serially diluted and plated on SBA plates to obtain the aerosol bacterial load concentration. The inhaled dose of spores (CFU/mL) was estimated using Guyton’s formula (64). Animals from each run were distributed equally among the different groups.

#### Mice

An inhalational mouse anthrax study was performed to assess the protection afforded by orally delivered prodrug sulopenem etzadroxil. Cohorts of female BALB/c mice (6–8 weeks, Charles River, Kingston, NY) were challenged by whole-body aerosol with spores of *B. anthracis* Ames as previously described. Mice received a mean inhaled dose of 15.3 × LD_50_ (range 13.6 to 16.9; 1 LD_50_ = 9.0 × 10^4^ CFU) in a whole-body exposure chamber. Antibiotic treatments were initiated at 24 h post-challenge and continued for 14 days. All treatments were given every 8 h. Treatment cohorts consisted of 10 mice per group. Disease progression and survival were assessed out to 34 days post-challenge. At the study termination, bacterial burden in surviving cohort lungs was assessed by serial dilution of tissue homogenate.

#### Rabbit

An inhalational rabbit study was performed to assess the efficacy of subcutaneous sulopenem. NZW rabbits (*N* = 6/group) were challenged with an average dose of 41.3 × LD_50_ (range 17.4 to 68.9; 1 LD_50_ = 1.05 × 10^5^ CFU) in a head-only exposure chamber. Treatment was initiated at 24 h post-challenge and continued for 14 days. All treatments were given q12h. Disease progression and survival were assessed out to 30 days post-challenge. At study termination, animals were humanely euthanized, and a terminal blood sample was taken to confirm sterility.

#### Statistics

Statistics for animal studies were calculated using GraphPad Prism software (GraphPad Software, La Jolla, CA). Differences in survival among the groups were determined by log-rank analysis of Kaplan-Meier survival curves.
